# Features of the immune status in patients with functional disorders of the gastrointestinal tract with non-psychotic mental disorders.

**DOI:** 10.1192/j.eurpsy.2023.2114

**Published:** 2023-07-19

**Authors:** N. P. Chernus, A. S. Sivkov, R. V. Gorenkov, S. I. Sivkov, ?. ?. Золотовицкая

**Affiliations:** 1 the Outpatient Care Department, the I.M. Sechenov First Moscow State Medical University, Москва; 2 the Department of Clinical Pharmacology and Internal Diseases Propaedeutics, Первый МГМУ им. И.М. Сеченова: Москва, Россия; 3 Scientific substantiation of health preservation of the population of the Russian Federation, The N. A. Semashko National Research Institute of Public Health; 4 Higher Healthcare Organization Management, the I.M. Sechenov First Moscow State Medical University, Москва; 5 the Department of Clinical Pharmacology and Internal Diseases Propaedeutics, the I.M. Sechenov First Moscow State Medical University, Moscow, Russian Federation

## Abstract

**Introduction:**

Актуальность исследования накопления данных о исключении иммунологического статуса у больных с выраженными расстройствами желудочно-кишечного тракта (ФЖКТ), характерных непсихотических заболеваний легких.

**Objectives:**

изучение взаимосвязи и появление признаков иммунологического и психовегетативного статуса больных у ФЖИР

**Methods:**

Проанализированы и рассчитаны результаты ПВА-обследования (тест MMPI) с иммунологическим статусом 57 больных (средний возраст 35,36 ± 14,43 года, мужчины-18, женщины-39) с ФАД в динамике с группой здоровых лиц 34 человека, среднего возраста 28 ,63±9,54 года

**Results:**

У больных с тревожной депрессией [средний профиль: пик по шкале 2 + ↑ 7-8 и ↓ 9 шкал] оценочное увеличение относительного значения Т-хелперов CD3+CD4+ (58,0 и 55,0% р = 0,015) при снижении относительного количества Т-цитотоксических лимфоцитов CD3+CD8+ (0,312 и 0,372, р=0,023; 14,0 и 19,0, р=0,001) соответственно, увеличение иммунорегуляторного индекса: Т-хелперы/Т-цитотоксические CD3+ CD4+/CD3+ CD8+ (4,143 и 2,600 , р=0,001); снижение относительного количества NK-клеток CD3-CD16+CD56+ (6% и 8%, р=0,01).

У больных с преобладанием соматизированной анестезии [ведущий пик по шкале 1]: основные выявлены в относительно сниженном CD3+ Т-лимфоцитов (53,0 и 60,0, р=0,001); снижение Т-хелперов: CD3+CD4+ (28,0% и 31,0%, р=0,001); повышение ЭКГ: CD3-СД16+СД56+ (34% и 26,0%, р=0,01; 1153 и 470, р=0,01) по сравнению со здоровыми людьми.

Выявленные изменения показателей иммунитета выявлялись в период обострения заболевания и носили транзиторный характер.

**Conclusions:**

Анализ данных с позиций клинико-динамического выявления вклада иммунобиологических регуляторов организма в клиническое затяжное течение и участие иммунной системы в механизме реализации психоэмоционального контроля стресса. .

**Disclosure of Interest:**

None Declared

